# Clinical index to quantify the 1-year risk for common postpartum mental disorders at the time of delivery (PMH CAREPLAN): development and internal validation

**DOI:** 10.1192/bjp.2023.74

**Published:** 2023-09

**Authors:** Simone N. Vigod, Natalie Urbach, Andrew Calzavara, Cindy-Lee Dennis, Andrea Gruneir, Brett D. Thombs, Mark Walker, Hilary K. Brown

**Affiliations:** Department of Psychiatry, Women's College Hospital and Research Institute, Toronto, Ontario, Canada; Department of Psychiatry, Faculty of Medicine, University of Toronto, Toronto, Ontario, Canada; ICES, Toronto, Ontario, Canada; and Institute for Health Policy, Management & Evaluation, University of Toronto, Toronto, Ontario, Canada; Faculty of Medicine, Queens University, Kingston, ON, Canada; ICES, Toronto, Ontario, Canada; Department of Psychiatry, Women's College Hospital and Research Institute, Toronto, Ontario, Canada; Department of Psychiatry, Faculty of Medicine, University of Toronto, Toronto, Ontario, Canada; and Lawrence S. Bloomberg Faculty of Nursing, University of Toronto, Toronto, Ontario, Canada; Institute for Clinical Evaluative Sciences, Toronto, Ontario, Canada; and Department of Family Medicine, University of Alberta, Edmonton, Alberta, Canada; Lady Davis Institute for Medical Research, Jewish General Hospital, Montreal, Quebec, Canada; Department of Psychiatry, McGill University, Montreal, Quebec, Canada; Department of Epidemiology, Biostatistics and Occupational Health, McGill University, Montreal, Quebec, Canada; Department of Medicine, McGill University, Montreal, Quebec, Canada; Department of Psychology McGill University, Montreal, Quebec, Canada; and Biomedical Ethics Unit, McGill University, Montreal, Quebec, Canada; Department of Obstetrics and Gynecology, Faculty of Medicine, University of Ottawa, Ottawa, Ontario, Canada; Department of Health & Society, University of Toronto Scarborough, Toronto, Ontario, Canada; Dalla Lana School of Public Health, University of Toronto, Toronto, Ontario, Canada; Women's College Research Institute, Women's College Hospital, Toronto, Ontario, Canada; and ICES, Toronto, Ontario, Canada

**Keywords:** Postpartum, depression, anxiety, predictive model, risk index

## Abstract

**Background:**

Common postpartum mental health (PMH) disorders such as depression and anxiety are preventable, but determining individual-level risk is difficult.

**Aims:**

To create and internally validate a clinical risk index for common PMH disorders.

**Method:**

Using population-based health administrative data in Ontario, Canada, comprising sociodemographic, clinical and health service variables easily collectible from hospital birth records, we developed and internally validated a predictive model for common PMH disorders and converted the final model into a risk index. We developed the model in 75% of the cohort (*n* = 152 362), validating it in the remaining 25% (*n* = 75 772).

**Results:**

The 1-year prevalence of common PMH disorders was 6.0%. Independently associated variables (forming the mnemonic PMH CAREPLAN) that made up the risk index were: (P) prenatal care provider; (M) mental health diagnosis history and medications during pregnancy; (H) psychiatric hospital admissions or emergency department visits; (C) conception type and complications; (A) apprehension of newborn by child services (newborn taken into care); (R) region of maternal origin; (E) extremes of gestational age at birth; (P) primary maternal language; (L) lactation intention; (A) maternal age; (N) number of prenatal visits. In the index (scored 0–39), 1-year common PMH disorder risk ranged from 1.5 to 40.5%. Discrimination (C-statistic) was 0.69 in development and validation samples; the 95% confidence interval of expected risk encompassed observed risk for all scores in development and validation samples, indicating adequate risk index calibration.

**Conclusions:**

Individual-level risk of developing a common postpartum mental health disorder can be estimated with data feasibly collectable from birth records. Next steps are external validation and evaluation of various cut-off scores for their utility in guiding postpartum individuals to interventions that reduce their risk of illness.

The postpartum period is a high-risk time for the development of a mental disorder. Depression, anxiety and related disorders (such as obsessive–compulsive disorder or post-traumatic stress disorders) are collectively the most common, with prevalence ranging from 7 to 20%, depending on the sample.^1–3^ These common postpartum mental health (PMH) disorders can impair activities of daily living such as eating and sleeping and have a negative impact on social and occupational functioning.^2^ Parent–infant interactions can also be affected, with implications for long-term socioemotional difficulties in children.^4,5^ Preventing and treating common PMH disorders is therefore a major public health priority.

Various psychological interventions can prevent common PMH disorders from developing, as can antidepressant use in individuals with a history of the illness.^2,6^ These interventions are most effective though among those at high risk; they may be minimally effective or ineffective in prevention in individuals who are at low risk.^2^ So, the ability to quantify a person's risk for developing a common PMH disorder has clinically actionable implications. For example, an individual-level estimate of risk could help with decisions about when to devote time (and/or financial resources) to psychotherapy, or when to recommend preventive antidepressant use.

Clinical prediction models can facilitate evidence-based clinical decision-making at the point of care.^7^ Many models have an associated points-based risk-scoring system – a clinical risk index – to allow for a rapid individual-level risk assessment.^8^ A clinical risk index does not aim to predict with absolute certainty whether the future event will occur. Rather, it provides a framework for intervention options to be recommended based on an individual's calculated risk for the outcome.^9^ Well-known clinical risk indices include the Framingham Risk Score (FRS) for heart disease and the Fracture Risk Assessment Tool (FRAX) for fragility fractures.^10,11^ Randomised controlled trials (RCTs) have shown that use of these indices can improve patient outcomes, and they are recommended globally by various clinical practice guidelines.^12^

Previous attempts to create risk indices for postpartum depression using key risk factors such as prior psychiatric illness and major stressors such as obstetrical and neonatal complications, financial and marital difficulties and inadequate social support^2^ have yielded moderate predictive capacity,^13,14^ similar to that of indices such as the FRAX and FRS.^10,11^ These indices have not been taken up into clinical practice, however, and so have not been examined to determine at what level of risk preventive intervention(s) might improve outcomes. This may be because the variables required to calculate these risk indices include patient self-reported life stressors, social support and subsyndromal depressive symptoms. This means that provider and patient time is required for data acquisition and entry at the point of care. No risk indices have been derived or validated at the population level, none have also been inclusive of other common PMH disorders, such as postpartum anxiety, and none have used only variables that could be automatically extracted from routinely collected information in a health record.

In a population-based cohort in Ontario, Canada's largest province for which data on common PMH disorder risk factors and diagnoses are routinely recorded in health administrative data-sets, we aimed to develop a clinical risk index that could estimate a postpartum person's specific risk for developing a common PMH disorder in the 1 year postpartum using data easily collectable at the time of birth and to conduct an internal validation of the risk index.

## Method

### Study design and data sources

This population-based cohort study leveraged linked health administrative and clinical registry data collected in Ontario, Canada, between 2012 and 2015. Ontario has a universal publicly funded health insurance system that includes obstetrical care and physician-based mental healthcare for all Ontario residents. Data were analysed at ICES (formerly the Institute for Clinical Evaluative Sciences), an independent non-profit research organisation that holds population-based data to analyse healthcare use and effectiveness in Ontario, and where patient records are de-identified and linked using unique encoded identifiers. ICES data-sets contain complete and accurate sociodemographic information, including age, neighbourhood-level income, immigration status, and physicians’ and hospital-based diagnoses (Supplementary Table 1, available at https://dx.doi.org/10.1192/bjp.2023.74)^15,16^ and are linked the Better Outcomes Registry Network (BORN), which uses the standardised BORN Information System (BIS) to collect clinical data on all Ontario births and has built-in checks for validity to verify the data at data entry.^17^ BIS data for each birth in 2012–2014 are linked to other health administrative data with a success rate of 93%.^18^

### Participants

Postpartum individuals (female based on their provincial health card) who delivered a live infant in or outside of hospital from 1 April 2012 to 31 March 2014 were identified. As the aim of the risk index was to predict a new or recurrent illness (i.e. one that could be prevented with intervention), not to identify those already in treatment for their conditions, those who had received care for any mental illness from a physician during the 2 years before delivery were excluded, as were those with psychotic disorders diagnosed from the earliest available data in the health administrative databases.^19^ For individuals with multiple deliveries during the study period, we randomly selected one delivery per person to contribute to the cohort, as opposed to selecting the first delivery during the study period, so as to avoid over-selection of primiparous deliveries into the cohort.

### Outcome

The primary outcome was a common mental health disorder diagnosed on a visit to a physician in an out-patient or in-patient setting during the first year postpartum.^2^ Eligible diagnoses were major depressive episodes (including those associated with major depressive disorder and bipolar disorder) and any anxiety or related disorder (e.g. obsessive–compulsive disorder or post-traumatic stress disorder) (Supplementary Table 2). We required: (a) two or more out-patient visits with a diagnosis of an eligible disorder (to avoid ‘rule-out’ visits) or (b) at least one hospital admission or emergency department visit with a diagnosis of an eligible disorder, during the outcome period. The sensitivity and specificity of identifying common mental health disorders in primary care are 80.7% and 97.0% respectively, compared with chart diagnoses (positive and negative predictive values 84.9% and 96.0% respectively).^20^

### Risk index variables

All variables were captured either in Ontario health administrative data or in Ontario's standardised antenatal reporting forms that make up part of the BIS data-set, to maximise potential for scale and spread of any risk index created. Variables were grouped as: (a) sociodemographic (maternal age, parity, neighbourhood income quintile, location of residence, primary language, immigration status); (b) pre-pregnancy (e.g. prior psychiatric diagnoses, hospital admissions); (c) pregnancy (smoking, alcohol or substance use during pregnancy, intimate partner violence in pregnancy, prenatal care provider type and number of prenatal visits, pregnancy complications); (d) labour and delivery; and (e) child (sex, number of fetuses, gestational age at birth, size for gestational age, Apgar score, newborn complications, skin-to-skin contact, breastfeeding, newborn taken into care (‘newborn apprehension’) by child welfare services after delivery). Missing data were modelled as levels in variable categories, as applicable. A full list of variables is given in Supplementary Table 3.

### Analysis

A split-sample methodology was used to create and then validate the predictive model. The cohort was randomly divided into two groups to create a larger ‘development’ data-set and a smaller ‘validation’ data-set, at a 2:1 ratio.

#### Model development

We first characterised all potential risk index variables and their relationships with the outcome, using logistic regression to generate odds ratios (OR) and 95% confidence intervals (CI). Next, we developed a predictive model by creating a series of logistic regression models in which potential risk index variables were sequentially added from each of the five variable groups described above.^21,22^ The log likelihood test (−2LL) determined whether added variables increased the model's predictive capacity compared with a simpler version beyond what would be expected with more variables. To build the most parsimonious model, variables were removed if the absolute effect size (parameter estimate) was <0.05 and their removal did not reduce the C-statistic by >0.001 or change the effect size of another covariate by >10%. C-statistics were generated to characterise model discrimination in both the development and validation data-sets.

We conducted two sets of additional analyses to finalise decisions about what model to convert into a risk index. First, replicating procedures from the primary analysis, separate models were built for (a) depressive episodes and (b) anxiety and related disorders. If individual diagnostic models outperformed the main model, we planned to create separate risk indices. Second, to determine whether an empirical machine learning approach would have improved predictive ability over the manual modelling approach, we entered all potential variables into a random forest classifier.^23^ If this were to substantially outperform the logistic modelling procedure, this might have additionally supported creating an online automated risk calculator system for jurisdictions where perinatal care data are routinely entered electronically. To conduct this analysis, a random forest classifier utilising the full data-set (development and validation combined, *n* = 228 134) was created using the *randomforest* package in R version 3.1.2 for Linux (2014). For each decision tree, observations were randomly sampled with replacement to form a training set (63.2% of the full data-set) and those not selected (out-of-bag sample) were used as a validation set. Predictor variables (Supplementary Table 3) were specified in the same way as in the logistic models, including categories for missing values, except maternal age, which was linear and continuous. The number of variables randomly sampled as candidates at each split in a decision tree was the square root of the number of variables in the data-set, rounded down (e.g. 12 variables in Model 5, containing all 5 variable groups). Trees were grown until the out-of-bag error rate stabilised to within 0.01%, ranging from 600 trees (Model 2) to 760 trees (Model 5). A receiver operating characteristic (ROC) curve and C-statistic (area under ROC curve) were computed for the out-of-bag sample using the *prediction* and *performance* functions of the ROCR package in R.

#### Risk index

Once the final model was established, we converted it into a risk index.^10^ There were several steps involved in this procedure. Using the regression coefficient estimates of the multivariable model, the variables were organised into categories so that we could assign a reference value (midpoints and distributions were examined to generate categorisations for continuous variable; we then calculated the distance of each category of a variable (in regression units) from the reference category. Finally, assigning the reference category a point value of 0, we computed a point value for every category. This conversion of model parameters into integers was done to create a situation where the risk index could be easily computed in multiple settings, including by pen and paper or a simple electronic calculator, and thus be applicable equitably in lower and higher resource settings.

We then generated for each score of the risk index: (a) the probability (or risk) for a common PMH disorder; (b) the expected and observed probabilities of the outcome, which if similar would indicate adequate calibration; and (c) the sensitivity, specificity and positive and negative predictive values if the score were to be used as a screening ‘cut-off’. Although we did not have an external sample in which to conduct validation, we conducted additional cross-validation. As Ontario is a large geographical area with community supports and health services organised regionally, generalisability of the model could differ by region. Socioeconomic status also varies across the Province. We therefore conducted a set of ‘leave-one-out’ analyses by maternal region of residence and income in the validation sample. To do this, we assigned individuals to one of five regions based on the first letter of their postal code (Eastern Ontario, Western Ontario, Northern Ontario, ‘Golden Horseshoe’ wrapping around Lake Ontario, and Toronto, Ontario) and to one of five neighbourhood income quintiles, which are defined by linkage of the first three postal code digits to income data from census files for that neighbourhood. We then conducted ‘leave-one-group-out’ analyses by region and separately by neighbourhood income quintile. For each fold, each listed group was iteratively left out of the model, and the ‘final model’ was fit to the remaining four groups. The fitted model was then used to predict the outcome in the left-out group.

With the exception of the machine learning analayses, all analyses were conducted using SAS^Ⓡ^ version 9.4 for Unix (2014).

## Results

From the 280 225 live births in Ontario during the study period, about 4.1% were excluded owing to invalid maternal identifiers for data linkage (*n* = 11 432), or inability to link maternal to infant data or maternal death before discharge from the delivery hospital admission (*n* = 76). Of the remaining 268 717, there were 16 846 (6.3%) births excluded owing to lack of Ontario Health Insurance Plan (OHIP) eligibility in the 2 years prior to delivery, which would have precluded measurement of pre-delivery covariates, and 79 (<0.1%) births excluded owing to non-Ontario residency, which would have precluded use of OHIP data to measure the PMH outcome. Additional exclusions were 1365 (0.5%) with severe mental illness at any time prior to index and 16 657 (6.2%) who had received mental healthcare in the 2 years before delivery. For the 5636 pregnancies with multiple deliveries during the study period, we randomly selected one per person, resulting in a final cohort of 228 134 women (*n* = 152 362 and *n* = 75 772 randomly assigned to the development and validation samples respectively).

About 6.0% (*n* = 13 608) of the cohort developed a common PMH disorder, including 3392 (1.5%) with a depressive episode, 11 262 (5.0%) with an anxiety or related disorder and 1046 (0.5%) with both. PMH disorders were usually first diagnosed in the out-patient setting (88.1%), followed by the emergency department (10.3%) or on hospital admission (1.6%). Those with common PMH disorders were slightly younger than those without (mean 29.9, s.d. = 5.8 *v*. 30.6, s.d. = 5.3 years), more likely to be primiparous (45.2% *v*. 42.0%) and less likely to be recent immigrants (16.7% *v*. 27.2%) (Table 1). They were more likely to report having previously experienced postpartum depression (4.4% *v*. 1.4%), any depression (15.3% *v*. 4.4%) and any anxiety disorder (13.8% *v*. 4.3%). They were more likely to report smoking (15.0% *v*. 10.2%), use of non-prescribed substances (3.0% *v*. 1.4%) and intimate partner violence in pregnancy (2.7% *v*. 1.5%). Pregnancy complications (16.0% *v*. 13.9%), preterm birth (8.2% *v*. 6.8%) and low Apgar scores (<7 at 1 or 5 min) (10.5% *v*. 7.6%) were also more common in this group.

**Table 1:** Selected characteristics of *n* = 228 134 individuals with and without a common postpartum mental health (PMH) disorder

	Common PMH disorder (*n* = 13608)	No common PMH disorder (*n* = 214 526)
Sociodemographics		
Maternal age at delivery, years: mean (s.d.)	29.9 (5.8)	30.6 (5.3)
Primiparous, *n* (%)	6149 (45.2)	90 053 (42.0)
Neighbourhood income quintile (Q1, lowest), *n* (%)	2945 (21.6)	43 274 (20.2)
Urban location of residence, *n* (%)	12 187 (89.6)	192 152 (89.6)
Primary language English, *n* (%)	11 079 (81.4)	166 976 (77.8)
Non-immigrant, *n* (%)	11 327 (83.2)	156 319 (72.9)
Pre-pregnancy mental health, *n* (%)		
Previous postpartum depression	598 (4.4)	3097 (1.4)
Lifetime history of anxiety	1877 (13.8)	9146 (4.3)
Lifetime history of depression	2079 (15.3)	9504 (4.4)
Lifetime history of substance use disorder	60 (0.4)	436 (0.2)
Lifetime psychiatric hospital admission or emergency department visit	1959 (14.4)	13 186 (6.1)
Pregnancy variables, *n* (%)		
Assisted conception	894 (6.6)	13 848 (6.5)
Smoking in pregnancy	2044 (15.0)	21 796 (10.2)
Alcohol use in pregnancy	1407 (10.3)	21 309 (9.9)
Illicit substance use in pregnancy^a^	406 (3.0)	2917 (1.4)
Intimate partner violence in pregnancy^b^	364 (2.7)	3292 (1.5)
Intention to breastfeed	11 407 (83.8)	185 234 (86.3)
Prenatal care provider (not mutually exclusive)		
Obstetrician, *n* (%)	10 368 (76.2)	160 780 (74.9)
Family physician, *n* (%)	3918 (28.8)	54 980 (25.6)
Midwife, *n* (%)	1634 (12.0)	29 309 (13.7)
Number of prenatal visits, mean (s.d.)	15.6 (6.7)	14.0 (6.5)
Any maternal pregnancy complication, *n* (%)^c^	2176 (16.0)	29 777 (13.9)
Labour and delivery variables, *n* (%)		
Any postpartum complication^d^	559 (4.1)	6310 (2.9)
Caesarean section	4015 (29.5)	58 413 (27.2)
Maternal ICU admission	50 (0.4)	516 (0.2)
Child variables, *n* (%)		
Child sex (female)	6696 (49.2)	105 250 (49.1)
Singleton	13 297 (97.7)	210 548 (98.1)
Gestational age at birth >37 weeks	12 350 (90.8)	199 996 (93.2)
Small for gestational age (<10th centile)	1229 (9.0)	18 501 (8.6)
Apgar score <7 at 1 or 5 min	1428 (10.5)	16 439 (7.6)
Any newborn complication^e^	1051 (7.7)	11 975 (5.6)
Neonatal ICU admission	2159 (15.9%)	26 182 (12.2)
Skin to skin immediately post-birth	5423 (39.9)	93 805 (43.7)
Latch achieved by discharge	6772 (49.8)	114 142 (53.2)
Breastfeeding support provided in hospital	6044 (44.4)	99 990 (46.6)
Newborn apprehended by social services^f^	36 (0.3)	176 (0.1)
Neonatal death in hospital	105 (0.8)	411 (0.2)

ICU, intensive care unit.

a. Cannabis, cocaine, gas, hallucinogen, methadone, narcotic, opioid, other.

b. Data missing for 58 187 (25.5%) women.

c. Hypertensive disorders of pregnancy (gestational pre-existing + pre-eclampsia; pre-eclampsia; eclampsia; haemolysis, elevated liver enzymes and low platelet count (HELLP syndrome)), diabetes mellitus in pregnancy (type 1, type 2, gestational diabetes\insulin, gestational diabetes\no insulin), hyperemesis gravidarum, anaemia, bleeding, preterm premature rupture of membranes (PPROM), PROM).

d. Late postpartum haemorrhage, uterine atony, hysterectomy, abdominal incision infection, perineal laceration, pulmonary embolus, mastitis, thrombophlebitis, urinary tract infection, postpartum fever, postpartum perineal hematoma.

e. Neonatal abstinence syndrome, congenital malformation, infection, intraventricular haemorrhage, persistent fetal circulation, respiratory distress syndrome, seizure, sepsis.

f. Apprehended denotes that the newborn is taken into the care of social services.

In the development sample (*n* = 152 362), predictive capacity was low when only sociodemographics were included (5 variables, C-statistic of 0.586), but improved with the addition of pre-pregnancy mental health (9 variables, C = 0.655) and pregnancy variables (15 variables, C = 0.685) (Supplementary Table 4). Adding labour/delivery and then child variables led to slight improvements in discriminative capacity, albeit with a large number of additional variables (20 variables, C = 0.687, and 23 variables, C = 0.690, respectively) (Supplementary Table 4). As more variables are not advantageous when the objective is to create a risk index that can be easily computed in most healthcare contexts, the final model was simplified to maintain its discriminative capacity with the minimum number of variables. This led to a final model of 16 variables (C = 0.688) (Table 2). The model had similar discriminative capacity in the validation data-set (C = 0.686).

**Table 2:** Balanced logistic regression model in the development cohort (*n* = 152 362) predicting common postpartum mental health disorder, with the 16 variables presented using fully adjusted odds ratios (OR) and 95% confidence intervals

Variable	Beta estimate	Standard error	OR (95% CI)
**Maternal age**			
Age	−0.381	0.084	
Age-squared function	0.010	0.003	
Age-cubed function	−8.85e10^−5^	3.12e10^−5^	
**Primary language**			
Other			1.00 (referent)
English	0.290	0.058	1.34 (1.19–1.50)
French	0.023	0.119	1.02 (0.81–1.29)
**Immigration status**			
Immigrant			1.00 (referent)
Non-immigrant or long-term resident	0.4459	0.032	1.56 (1.47–1.66)
**Mental health diagnoses (for each, referent 1.00 = 'no' for disorder)**			
Postpartum depression history	0.803	0.060	2.23 (1.99, 2.51)
Lifetime anxiety disorder	0.771	0.037	2.16 (2.01, 2.32)
Lifetime depressive disorder	0.901	0.036	2.46 (2.30, 2.64)
Lifetime bipolar disorder	0.941	0.165	2.56 (1.86, 3.54)
Lifetime other mental disorder	0.665	0.115	1.94 (1.55, 2.44)
**Prior psychiatric hospital admission or emergency department visit**	0.512	0.035	1.67 (1.56, 1.79)
**Conception type**			
No assisted conception			1.00 (referent)
Assisted conception	0.227	0.060	1.25 (1.12, 1.41)
**Antenatal care provider (for each, referent 1.00 = ‘no’ for provider)**			
Family physician	0.052	0.030	1.05 (0.99, 1.12)
Midwife	0.320	0.049	1.38 (1.25, 1.52)
Nurse practitioner	−0.506	0.143	0.60 (0.46, 0.80)
Obstetrician	−0.042	0.037	0.96 (0.89, 1.03)
Other care provider	−0.179	0.096	0.84 (0.69, 1.01)
No antenatal care provider	0.031	0.153	1.03 (0.77, 1.39)
**Prenatal visits (per visit)**	0.056	0.002	1.06 (1.05, 1.06)
**Preterm premature rupture of membranes (yes)**	0.191	0.081	1.21 (1.03, 1.42)
**Any postpartum complication (yes)**	0.266	0.056	1.31 (1.17, 1.46)
**Neonatal abstinence syndrome (yes)**	0.565	0.191	1.76 (1.21, 2.56)
**Neonatal death in hospital (yes)**	0.984	0.188	2.68 (1.85, 3.87)
**Medications (for each, referent 1.00 = ‘no’ for specified medication)**			
Antiemetic	0.165	0.047	1.18 (1.08, 1.29)
Opioid	0.200	0.114	1.22 (0.98, 1.53)
**Gestational age**			
Term (37 weeks or more)			1.00 (referent)
20–23 weeks	0.803	0.256	2.23 (1.35, 3.68)
24–31 weeks	0.686	0.095	1.99 (1.65, 2.39)
32–36 weeks	0.179	0.046	1.20 (1.09, 1.31)
**Lactation intention**			
Yes			1.00 (referent)
No	0.228	0.040	1.26 (1.16-1.36)
**Apprehension of newborn by child protective services**			
No			1.00 (referent)
Yes	0.598	0.242	1.82 (1.13, 2.92)

Random forest model results yielded only slightly increased discriminative capacity in the validation sample compared with the clinical risk index (Supplementary Table 5). Logistic regression models constructed specifically for depressive episodes yielded a higher discriminative capacity in the development sample (C = 0.733) but this increase in discriminative capacity was not borne out in the validation sample (C = 0.698). Models constructed specifically for anxiety and related disorders yielded discriminative capacity similar to that observed in the main models (development C = 0.683; validation C = 0.680). Since neither the random forest model nor the diagnosis-specific models had improved performance, we retained the main model for the risk index.

**Fig. 1:**
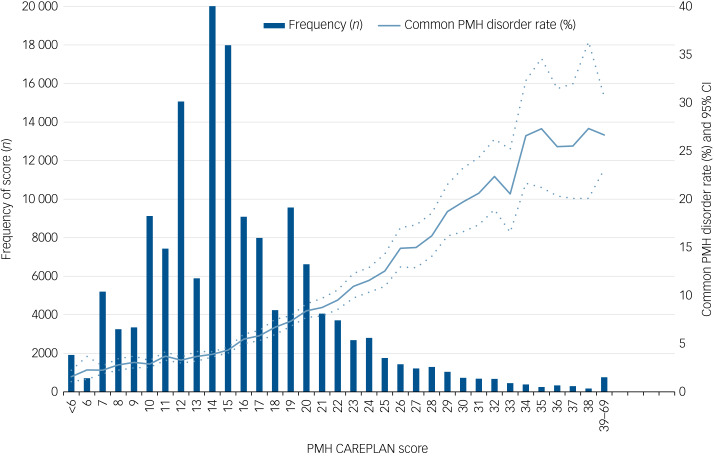
Distribution of risk scores in the development cohort (*n* = 152 362) and observed common postpartum mental health (PMH) disorder rate and 95% confidence interval (CI) associated with each point on the risk index.

When converting the final model into a risk index, we created the acronym ‘PMH CAREPLAN’ as a useful mnemonic for remembering its variables in the risk index that potentially would be used to plan an individual's PMH care: (P) prenatal care provider; (M) mental health diagnosis history and medications during pregnancy; (H) psychiatric hospital admissions or emergency department visits; (C) conception type and complications; (A) apprehension of newborn by child services; (R) region of maternal origin; (E) extremes of gestational age at birth; (P) primary maternal language; (L) lactation intention; (A) maternal age (at delivery); and (N) number of prenatal visits (Table 3). As very few observations occurred at the extremes and estimates of outcome risk were unstable below 0 and over 39, scores were left- and right-trimmed to 0 and 39. Expected probability of a common PMH disorder using the PMH CAREPLAN index ranged from 1.56% at a score of less than 6 to 40.5% at a score of 39 or above, and the relationship appeared linear (Fig. 1).

**Table 3:** PMH CAREPLAN index (range 0 to 39) for risk of common postpartum mental health disorders, with points assigned to values within the 16 variables in the index

Abbreviation	Risk factor	Value	Point value
P	Prenatal care provider	Obstetrician	0
		Family physician	1
		Midwife	3
		Nurse practitioner	−5
		Other	−2
M	Mental health diagnosis history (lifetime)	No prior diagnosis	0
		Postpartum depression	8
		Anxiety	8
		Depression	9
		Bipolar disorder	9
		Other	7
	Medications during pregnancy	Anti-emetic	2
		Opioid	2
H	Psychiatric hospital admissions or emergency department visits	None	0
		Lifetime	5
C	Conception type	Spontaneous conception	2
	Complications	Preterm premature rupture of membranes	2
		Any postpartum complication^a^	3
		Neonatal abstinence syndrome	6
		Neonatal death in hospital	10
A	Apprehension of newborn by child services (at birth)^b^	No apprehension	0
		During the delivery hospital admission	6
R	Region of maternal origin	Immigrant	0
		Non-immigrant	4
		Primary language French or other	0
		Primary language English	3
E	Extremes of gestational age at birth	37+	0
		32–36	2
		24–31	7
		20–23	8
P	Primary maternal language	French or other	0
		English	3
	
	
L	Lactation intention	Intending to breastfeed	0
		Not intending to breastfeed	2
A	Maternal age (at delivery)	26–38	0
		24–25, or 39 or older	1
		22–23	2
		20–21	3
		19	4
		18	5
		Under 18	7
N	Number of prenatal visits	0–8	0
		9–17	5
		18–26	10
		27+	15
	Total		
	If <0 → 0, if >39 → 39		
	Final total		

a. Postpartum complications are: late postpartum haemorrhage, uterine atony, hysterectomy, abdominal incision infection, perineal laceration, pulmonary embolus, mastitis, thrombophlebitis, urinary tract infection, postpartum fever, postpartum perineal hematoma.

b. The newborn is taken into the care of social services.

The expected probability of a common PMH disorder was within the 95% confidence interval observed for all scores in both samples (except for a score of 39 or above, where the expected rate was above the 95% confidence interval of the observed probability), indicating adequate calibration (Supplementary Table 6). In the validation sample, when the scores were treated as screening cut-offs, the best ‘trade-off’ of sensitivity/specificity (0.620/0.654) appeared to be at a threshold score of 17 or above. The higher the cut-off score (or threshold), the greater the specificity and the lower the sensitivity; given the low overall risk, almost all thresholds were associated with low positive predictive values and high negative predictive values (examples of various cut-off scores for both the development and validation samples are in Supplementary Table 7). In the leave-one-out analyses, discriminative capacity ranged from 0.65 to 0.71 and 0.67 to 0.69 based on provincial region of residence and the neighbourhood income quintile respectively (Supplementary Table 8).

## Discussion

We developed and internally validated a predictive model quantifying the risk for receiving a diagnosis of a common mental health disorder in the first postpartum year in a population-based sample. The model approached 70% in its ability to discriminate between those who would or would not develop a common PMH disorder over their first year postpartum, a discriminative capacity that was reasonably robust to leave-one-out analyses based on maternal region of residence and income level. The PMH CAREPLAN is the first published risk score in this area that considers all common PMH disorders, not only depressive episodes. It is also the first to use a physician-based clinical diagnosis, where outcome specificity is likely to be greater than that of prior risk indices where a diagnosis was assigned based on a cut-off on a self-report symptom scale.^24^

### Strengths and limitations

The model had similar discriminative capacity to previous studies, all of which predicted postpartum depression specifically (e.g. C = 0.71 in validation studies^13,14^ and C = 0.77 in a development-only sample^24^), and were survey-based and required depressive symptom scale input early postpartum. The predictive capacity of the current model could perhaps be improved by including both routinely collected and self-report variables that provide more information on clinical symptomatology and on the social context. For example, certain risk factors were not measurable in our data-sets and would have to be collected by self-report, such as subsyndromal symptoms and psychosocial stressors (e.g. life stress, low social support).^4^ Further, knowledge about certain postnatal factors might have improved predictive capacity, such as length of parental leave, nature of social support over time or other factors that occur postnatally that would not have been known at the time of delivery. However, advantages of the current model are that it generates a reasonable estimate of risk with routinely collected clinical data alone and that it uses data that are available at the time of delivery, which is an opportune time for identifying individuals who may benefit from additional monitoring or targeted intervention, given that all individuals are in contact with healthcare professionals.

### Clinical and research implications

Although improving predictive capacity is a laudable goal, most risk indices do not rely on perfect prediction of a future state for actionable results.^11,25^ For example, clinical practice guidelines recommend preventive treatment with osteoporosis medications when the 10-year risks of a major fracture or hip fracture exceed 20% and 3% respectively based on the FRAX index.^11^ In a trial of FRAX screening, individuals were offered preventive treatment with osteoporosis medications based on their 10-year risk level; in the screened group preventive treatment uptake was much higher and risk of hip fracture was reduced by 28%, lending support to the use of the index.^12^ With PMH CAREPLAN, one could risk stratify based on the 1-year risk for a common PMH disorder to develop, and evaluate evidence-based psychological or pharmacological interventions accordingly for whether screening with the risk index improves the uptake of preventive interventions and reduces the risk for a PMH disorder to develop. One could also test interventions based on a cut-off or threshold level of risk. Where the threshold risk level is set for an intervention would depend on tolerance for missing cases and/or for false-positive results. At the cut-off score of 17 or above that optimises the balance between sensitivity and specificity, about 62% of cases would be identified, with a false-positive rate of about 35%. This is likely to be a reasonable threshold for evaluation of monitoring strategies or of lower-cost preventive interventions with low potential for harm (i.e. where it is not highly problematic if some people receive the intervention who may not have needed it). In contrast, a higher cut-off score optimising specificity might be more appropriate for evaluating interventions with more potential for adverse effects (e.g. medications).

Prior to testing interventions, however, external validation is recommended. Although the prevalence of common PMH disorders (~6%) in this study is consistent with previous population-level estimates, using health administrative data limited our ability to capture individuals who did not come to medical attention. This means that the model could perform differently when diagnoses are ascertained systematically for an entire population. This might be particularly important because we found that being a recent immigrant and not speaking English as the primary language were protective factors in our model. Although this may reflect a healthy immigrant effect and/or the protective nature of postpartum cultural rituals that increase social support, the cultural and language barriers faced by immigrants can increase risk for common PMH disorders and reduce access to healthcare.^26,27^ It is also important to note that the PMH CAREPLAN risk index focuses on risk for common postpartum mental disorders (depressive, anxiety and related disorders such as obsessive–compulsive and trauma- and stressor-related disorders).^2^ These common PMH disorders are to be distinguished from the more rare but severe postpartum mental disorders such as postpartum psychosis, bipolar disorder and primary psychotic disorders, whose distinct clinical, epidemiological and biological risk factors would likely necessitate a separate risk index building process.^28^

### Summary and next steps

The PMH CAREPLAN risk index creates a framework for clinically actionable risk stratification that could assist patients and providers in determining an individual's level of risk for common postpartum mental health disorders and direct them to appropriate intervention. After evaluation of its utility at optimising the uptake of preventive treatments and reducing risk for developing a common PMH disorder, it could be easily integrated into almost any type of antenatal or postpartum care setting. Future research to externally validate and refine the PMH CAREPLAN index and to explore the effectiveness of preventive interventions at varying levels of risk will be critical to its use in the clinical setting.

## Data Availability

The data for this study are not publicly available owing to restrictions related to the Personal Health Information Privacy Act of Ontario in its permissions for data use. However, detailed data-set creation and analytic plans (including health administrative diagnostic codes used and SAS^®^ coding) are available from the corresponding author on request.

## Supplementary material

For supplementary material accompanying this paper visit https://doi.org/10.1192/bjp.2023.74. click here to view supplementary material
