# Supplementary material for: Clinical index to quantify the 1-year risk for common postpartum mental disorders at the time of delivery (PMH CAREPLAN): development and internal validation
Source: Br J Psychiatry. 2023 Sep;223(3):422–9. doi: 10.1192/bjp.2023.74 (PMC10895501; doi:10.1192/bjp.2023.74)
Supplement: Supplementary file 1 [file S0007125023000740sup001.docx]

**Table S1. ICES Data Sources Used in the Current Study**

| (1) Registered Persons Database (RPDB) for age, sex, and postal code  (2) Ontario Health Insurance Plan (OHIP) for outpatient physician services (1992-present)  (3) National Ambulatory Care Reporting System (NACRS) for emergency department (ED) visits (2002-present)  (4) Discharge Abstract Database (DAD) for details about non-psychiatric hospitalizations, including data on obstetrical deliveries (MOMBABY dataset) and psychiatric hospitalizations before 2006 (1988-present)  (5) Ontario Mental Health Reporting System (OMHRS) for details about psychiatric hospitalizations (2006 -present)  (6) Immigration, Refugee and Citizenship Canada Permanent Resident Database (1985-present) for details about immigration status |
| --- |

**Table S2. Codes to identify common PMH disorders**

| **Hospital and Emergency Department (ICD-10)** | |
| --- | --- |
| F320-23,28 | Depressive episodes |
| F330-34,38-9 | Recurrent depressive disorder |
| F313-15 | Bipolar affective disorder, current episode depression |
| F411 | Generalized anxiety disorder |
| F410 | Panic disorder [episodic paroxysmal anxiety] |
| F400 | Agoraphobia |
| F420-22, 28-29 | Obsessive-compulsive disorders |
| F430 | Acute stress reaction |
| F431 | Post-traumatic stress disorder |
| F401 | Social phobias |
| F402, 08-9 | Specific (isolated) phobias, other phobic anxiety disorders |
| F412-13, 18-19 | Mixed anxiety/depressive disorders, other specified/unspecified anxiety |
| F530 | Mild mental and behavioural disorders associated with the puerperium, not elsewhere classified |
| **Hospital only (DSM Code)** | |
| 29620-26 | Major depressive disorder, single episode |
| 29630-36 | Major depressive disorder, recurrent |
| 29650-56 | Bipolar I disorder, most recent episode depressed |
| 30002 | Generalized anxiety disorder |
| 30001,21 | Panic disorder (with and without Agoraphobia) |
| 30022 | Agoraphobia without history of panic disorder |
| 30030 | Obsessive-compulsive disorder |
| 30830 | Acute stress disorder |
| 30981 | Post-traumatic stress disorder |
| 30023 | Social phobia |
| 30029 | Specific phobia |
| 30000 | Anxiety disorder, not otherwise specific |
| 30040 | Dysthymic disorder |
| **Outpatient physician diagnostic codes (Ontario Health Insurance Plan)** | |
| 296 | Bipolar disorder, severe depression |
| 300 | Anxiety disorders, obsessive compulsive disorder, posttraumatic stress disorder |
| 311 | Depressive disorders, not otherwise specified |

**Table S3. Full variable list**

| Study Variable |
| --- |
| 1. Socio-demographics |
| Maternal Age at Delivery |
| Parity |
| Neighbourhood Income (in quintiles, based on postal code of residence) |
| Location of Residence (urban = > 10,000/sq km) |
| Maternal primary language |
| Immigrant Status |
| *2.* Baseline Maternal Health Information |
| Maternal Psychiatric History (self-reported) lifetime history |
| Postpartum depression |
| Depression |
| Anxiety |
| Alcohol or substance use disorder |
| Bipolar disorder |
| Psychotic disorder |
| Psychiatric hospitalization or ED visit (2 or more years prior to delivery) |
| Maternal pre-pregnancy chronic conditions |
| Diabetes Mellitus |
| Hypertension |
| Autoimmune diseases |
| Cancer |
| Cardiovascular disease |
| Cerebrovascular disease |
| Congenital heart disease |
| Developmental delay |
| Endocrine disorders (hyper and hypothyroidism included as independent variables) |
| Gastrointestinal disorders |
| Hepatitis |
| Hematological disorders |
| Musculoskeletal disorders |
| Neurological disorders (epilepsy as independent variable) |
| Pulmonary disorders (asthma and pulmonary embolus as independent variables) |
| Urological conditions |
| Recurrent spontaneous abortions |
| 3.       Pregnancy-related health information |
| Behavioural Risk Factors |
| BMI (at onset of pregnancy, weight gain during pregnancy) |
| Smoking |
| Alcohol use |
| Illicit substance use (cannabis, cocaine, gas, hallucinogen, narcotic/opioid) |
| Prescribed medications |
| Intimate partner violence |
| Conception type (spontaneous, vaginal insemination, ovulation induction, intrauterine insemination, in-vitro fertilization) |
| Antenatal care provision |
| Provider (Obstetrician, family physician, midwife, nurse practitioner, other) |
| Prenatal physician visit number |
| Prenatal ultrasound first 20 weeks |
| Pregnancy complications |
| Hypertensive disorders of pregnancy (gestational pre-existing + pre-eclampsia, pre-eclampsia, eclampsia, HELLP) |
| Diabetes mellitus in Pregnancy (Type I, Type II, gestational diabetes\insulin, gestational diabetes\no insulin) |
| Other (hyperemesis gravidarum, anemia, bleeding, preterm premature rupture of membranes (PPROM), PROM) |
| Placental complications (abruption, increta, accrete, percreta, previa, other) |
| 4.       Labour and Delivery |
| Type of delivery (spontaneous or assisted, vaginal, caesarean (labour/no labour) |
| Provider attending birth (Obstetrician, family physician, midwife, nurse practitioner, other) |
| Episiotomy (mediolateral, midline) |
| Perineal Lacerations (1^st^, 2^nd^, 3^rd^, 4^th^, cervical tears) |
| Abnormal fetal surveillance |
| Meconium |
| Cord prolapse |
| Shoulder dystocia |
| Fever |
| Non-progressive first stage of labour |
| Non-progressive second stage of labour |
| Uterine Rupture |
| Pulmonary embolism |
| Hysterectomy |
| Placental abruption |
| Uterine Dehiscence |
| Placental abruption |
| Retained Placenta (surgical removal) |
| Retained Placenta (manual removal) |
| Perineal Hematoma |
| Postpartum hemorrhage |
| Uterine Atony |
| Postpartum complications |
| Late postpartum hemorrhage |
| Uterine Atony |
| Hysterectomy |
| Abdominal incision infection |
| Perineal infection |
| Pulmonary embolus |
| Mastitis |
| Thrombophlebitis |
| Urinary tract infection |
| Postpartum fever |
| Postpartum perineal hematoma |
| Maternal Intensive Care Unit (ICU) admission in pregnancy or postnatally |
| 5. Child Variables |
| Number of fetuses |
| Child Sex |
| Gestational Age at Birth |
| Birthweight |
| LGA (> 90th centile) |
| SGA (< 10th centile) |
| Apgar Score (1 and 5 minutes) |
| Complications |
| Birth injuries |
| Respiratory distress syndrome |
| Seizure |
| Sepsis |
| Intraventricular hemorrhage |
| Persistent fetal circulation |
| Neonatal abstinence syndrome |
| Congenital malformation |
| Neonatal intensive care unit (NICU) admission |
| Newborn death (in hospital) |
| Skin-to-skin and breastfeeding |
| Intention to breastfeed |
| Skin to skin contact (immediately post-birth with birth mother, anytime in hospital postbirth) |
| Attempt or opportunity to breastfeed in first 2 hours (for any reason) |
| Latch achieved (in birthing room, by discharge) |
| Latch or hand expression or pumping breastmilk by discharge |
| Breastfeeding support provided (assistant, includes lactation consult) |
| Newborn apprehended in the delivery hospitalization |

**Table S4.** Variables significant in successive logistic regression models adding in covariates by block in the derivation cohort (n=152,362, with discriminative capacity (c-statistic) and number of variables for each model

|  | Model 1 | Model 2 | Model 3 | Model 4 | Model 5 | Simplified Model 5 |
| --- | --- | --- | --- | --- | --- | --- |
| Socio-demographics | Maternal age  Primary language  Immigration status  Rural residence  Income | Maternal age  Primary language Immigration status  Rural residence | Maternal age  Primary language  Immigration status  Rural residence | Maternal age  Primary language  Immigration status  Rural residence | Maternal age  Primary language  Immigration status  Rural residence | Maternal age  Primary language  Immigration status |
| Pre-pregnancy health variables |  | Prior psychiatric diagnoses  Psychiatric hospitalization  BMI  Cardiac condition  Parity | Prior psychiatric diagnoses  Psychiatric hospitalization  BMI | Prior psychiatric diagnoses  Psychiatric hospitalization  BMI | Prior psychiatric diagnoses  Psychiatric hospitalization  BMI | Prior psychiatric diagnoses  Psychiatric hospitalization |
| Pregnancy-related variables |  |  | Prenatal care provider Number of prenatal visits  PPROM  Medications  Assisted conception  Smoking  Prior preterm labour  ICU admission | Prenatal care provider Number of prenatal visits  PPROM  Medications  Assisted conception  Smoking  Prior preterm labour  ICU admission | Prenatal care provider  Number of prenatal visits  PPROM  Medications  Assisted conception  Smoking | Prenatal care provider  Number of prenatal visits  PPROM  Medications  Assisted conception |
| Labour and Delivery variables |  |  |  | Delivery type  Episiotomy/Perinatal laceration  Any postpartum complication  Abruption  Provider attending birth | Delivery type  Episiotomy/Perinatal laceration  Any postpartum complication | Any postpartum complication |
| Child variables |  |  |  |  | Gestational age  Neonatal abstinence syndrome  Newborn death  Newborn apprehension Intention to breastfeed  Apgar scores  Achieving latch in hospital | Gestational age  Neonatal abstinence syndrome  Newborn death  Newborn apprehension Intention to breastfeed |
|  |  |  |  |  |  |  |
| # of variables | 5 | 9 | 15 | 20 | 23 | 16 |
| C-Statistic | 0.586 | 0.655 | 0.685 | 0.687 | 0.690 | 0.688 |

| Table S5. Random forest classifier c-statistics (validation sample) |  |  |  |
| --- | --- | --- | --- |
|  | **Outcome** | | |
| Model | **Any PMH Disorder** | **Depressive Disorder** | **Anxiety and Related Disorders** |
| 2 – Socio-demographics + Pre-pregnancy factors | 0.678 |  |  |
| 3 – Socio-demographics + Pre-pregnancy factors + Pregnancy | 0.707 |  |  |
| 4 – Socio-demographics + Pre-pregnancy factors + Pregnancy + Delivery | 0.712 | 0.706 | 0.703 |
| 5 – Socio-demographics + Pre-pregnancy factors + Delivery + Child | 0.720 | 0.711 | 0.713 |

**Table S6.** Expected probability of PMH compared to observed probability (and 95% confidence intervals, CI) for all scores in the development and validation samples

|  |  | Development Cohort  N = 152,362 | | | Validation Cohort  N = 75.772 | | |
| --- | --- | --- | --- | --- | --- | --- | --- |
| Risk Score | **Score-based Expected risk** | **Observed Rate** | **Lower 95 % CI** | **Upper 95% CI** | **Observed Rate** | **Lower 95 % CI** | **Upper 95% CI** |
| < 6 | 1.56 | 1.57 | 1.06 | 2.24 | 1.12 | 0.56 | 2.00 |
| 6 | 1.87 | 2.27 | 1.30 | 3.68 | 3.24 | 1.55 | 5.95 |
| 7 | 2.07 | 2.25 | 1.86 | 2.70 | 2.39 | 1.83 | 3.07 |
| 8 | 2.28 | 2.80 | 2.25 | 3.44 | 2.41 | 1.71 | 3.29 |
| 9 | 2.51 | 3.05 | 2.49 | 3.70 | 3.01 | 2.24 | 3.95 |
| 10 | 2.77 | 2.88 | 2.54 | 3.25 | 2.54 | 2.10 | 3.05 |
| 11 | 3.05 | 3.70 | 3.28 | 4.16 | 3.20 | 2.65 | 3.83 |
| 12 | 3.36 | 3.29 | 3.00 | 3.59 | 3.24 | 2.85 | 3.67 |
| 13 | 3.70 | 3.67 | 3.19 | 4.19 | 4.18 | 3.47 | 4.99 |
| 14 | 4.07 | 3.89 | 3.62 | 4.17 | 4.48 | 4.08 | 4.92 |
| 15 | 4.48 | 4.33 | 4.03 | 4.64 | 4.03 | 3.63 | 4.47 |
| 16 | 4.93 | 5.47 | 5.00 | 5.97 | 5.53 | 4.87 | 6.25 |
| 17 | 5.42 | 5.82 | 5.31 | 6.38 | 6.37 | 5.61 | 7.21 |
| 18 | 5.96 | 6.70 | 5.95 | 7.53 | 6.16 | 5.14 | 7.31 |
| 19 | 6.54 | 7.33 | 6.80 | 7.89 | 7.24 | 6.49 | 8.04 |
| 20 | 7.18 | 8.41 | 7.73 | 9.14 | 7.04 | 6.14 | 8.02 |
| 21 | 7.88 | 8.75 | 7.87 | 9.71 | 9.30 | 8.03 | 10.70 |
| 22 | 8.64 | 9.52 | 8.55 | 10.57 | 9.68 | 8.32 | 11.21 |
| 23 | 9.46 | 10.95 | 9.74 | 12.28 | 12.05 | 10.28 | 14.04 |
| 24 | 10.35 | 11.57 | 10.34 | 12.90 | 11.87 | 10.11 | 13.84 |
| 25 | 11.32 | 12.54 | 10.94 | 14.31 | 11.49 | 9.32 | 14.02 |
| 26 | 12.36 | 14.90 | 12.96 | 17.04 | 15.04 | 12.31 | 18.18 |
| 27 | 13.48 | 14.98 | 12.88 | 17.32 | 14.29 | 11.56 | 17.46 |
| 28 | 14.69 | 16.20 | 14.08 | 18.55 | 15.36 | 12.32 | 18.92 |
| 29 | 15.99 | 18.71 | 16.17 | 21.53 | 14.82 | 11.66 | 18.58 |
| 30 | 17.38 | 19.72 | 16.62 | 23.23 | 19.23 | 14.84 | 24.51 |
| 31 | 18.86 | 20.62 | 17.34 | 24.33 | 21.70 | 17.04 | 27.24 |
| 32 | 20.44 | 22.35 | 18.92 | 26.23 | 21.50 | 16.72 | 27.20 |
| 33 | 22.12 | 20.54 | 16.55 | 25.19 | 23.50 | 17.71 | 30.59 |
| 34 | 23.89 | 26.58 | 21.65 | 32.30 | 23.19 | 17.10 | 30.74 |
| 35 | 25.75 | 27.31 | 21.21 | 34.62 | 23.08 | 15.89 | 32.41 |
| 36 | 27.71 | 25.45 | 20.33 | 31.47 | 20.77 | 14.69 | 28.50 |
| 37 | 29.76 | 25.51 | 20.07 | 31.98 | 25.00 | 16.87 | 35.69 |
| 38 | 31.89 | 27.33 | 20.08 | 36.34 | 26.61 | 17.82 | 38.21 |
| 39-69 | 40.50 | 26.66 | 23.10 | 30.61 | 28.66 | 23.21 | 34.99 |

**Table S7.** Test characteristics for various PMH CAREPLAN cut-off scores in development and validation samples, including at optimal sensitivity and specificity of a threshold of 17 or above.

|  |  | Development Cohort (n=152,362) | | | | Validation Cohort (n=75,772) | | | |
| --- | --- | --- | --- | --- | --- | --- | --- | --- | --- |
| Cut-off Score | **Risk** | **Sensitivity** | **Specificity** | **Positive Predictive Value** | **Negative Predictive Value** | **Sensitivity** | **Specificity** | **Positive Predictive Value** | **Negative Predictive Value** |
| > 10 | 2.8% | 0.969 | 0.090 | 0.064 | 0.979 | 0.969 | 0.092 | 0.063 | 0.979 |
| > 15 | 4.5% | 0.774 | 0.456 | 0.083 | 0.969 | 0.773 | 0.457 | 0.082 | 0.970 |
| > 17 | 5.4% | 0.622 | 0.653 | 0.102 | 0.964 | 0.620 | 0.654 | 0.101 | 0.965 |
| > 20 | 7.2% | 0.442 | 0.820 | 0.135 | 0.958 | 0.435 | 0.820 | 0.132 | 0.958 |
| > 25 | 11.3% | 0.237 | 0.933 | 0.183 | 0.950 | 0.237 | 0.934 | 0.183 | 0.951 |
| > 30 | 17.4% | 0.125 | 0.974 | 0.234 | 0.946 | 0.122 | 0.974 | 0.227 | 0.946 |
| > 35 | 25.8% | 0.052 | 0.991 | 0.258 | 0.943 | 0.053 | 0.991 | 0.260 | 0.943 |

**Table S8. Cross-validation “leave-one-group-out” analyses where the original model was fit leaving out one group from the development sample, and then validated in the left-out group in the validation sample (n=75,370*).** Discriminative capacity is presented using c-statistics.

|  |  | Outcome | | |
| --- | --- | --- | --- | --- |
| Model – Group left out of model in development sample | **No. of participants in Region/Income Quintile in validation sample (N)** | **Any PMH Disorder**  **(c-statistic)** | **Depressive Disorder**  **(c-statistic)** | **Anxiety and Related Disorders**  **(c-statistic)** |
| Ontario Region |  |  |  |  |
| 1 – Eastern Ontario Region | 11,130 | 0.698 | 0.677 | 0.694 |
| 2 – Western Ontario Region | 14,825 | 0.696 | 0.730 | 0.688 |
| 3 – Northern Ontario Region | 4,344 | 0.713 | 0.703 | 0.710 |
| 4 – “Golden Horseshoe” Ontario Region | 29,566 | 0.669 | 0.657 | 0.665 |
| 5 –Toronto, Ontario Region | 15,505 | 0.647 | 0.632 | 0.648 |
| Neighbourhood Income Quintile |  |  |  |  |
| 1 – Lowest income quintile (Q1) | 15,355 | 0.696 | 0.708 | 0.688 |
| 2 – Q2 | 14,930 | 0.686 | 0.708 | 0.671 |
| 3 – Q3 | 15,470 | 0.680 | 0.685 | 0.681 |
| 4 – Q4 | 16,791 | 0.680 | 0.663 | 0.680 |
| 5 – Highest income quintile (Q5) | 12,824 | 0.670 | 0.682 | 0.665 |

*There were 403 individuals who had missing data on the above variables, and were excluded from this analysis.
